# Buildings Contemplated

**Published:** 1914-03-14

**Authors:** 


### Buildings Contemplated.

Aberdeen.—The Corporation Public Health Committee have agreed to advertise for a site for a sanatorium.

Bury.—The Guardians have resolved to obtain tenders for alterations at the workhouse. The plans are by Mr. Hopkinson.

Cefncoed.—The estimated cost of the proposed new mental hospital at Cefncoed, including foundations, superstructure, architect's commission, and cost of heating, machinery, etc., is £180,000. Tenders will shortly be obtained by the Swansea and Merthyr Joint Asylums Committee at Swansea for the foundations.

Denbigh.—North Wales Lunatic Asylum Committee are inviting tenders for the erection of a sanitary annexe and for extensions of the day rooms. The architect is Mr. William T. Lockwood, F.R.I.B.A., of 88 Foregate Street, Chester, from whom particulars may be obtained upon payment of a deposit of £3 3s.

Durham.—Alterations and extensions are to be carried out at the county hospital, at an estimated cost of £4,500.

East Ham.—The Town Council have decided to build a diphtheria block at the isolation hospital, at an estimated cost of £5,200.

Fairwood Common.—Tenders are being invited for the erection of an isolation hospital on Fairwood Common, near Upper Killag, Swansea. The architect is Mr. H. A. Ellis, 10 Fisher Street, Swansea, from whom particulars may be obtained.

Guisborough.—The Rural District Council have decided to ask the Redcar Council to co-operate with the surrounding rural parishes in providing an up-to-date hospital.

Hemel Hempstead.—The Joint Isolation Hospital Board has been authorised by the Local Government Board to borrow £5,687 for a new hospital for infectious cases.

Hendrefoilan.—The Swansea and District Sanatorium Committee are about to obtain tenders for a new hospital at Hendrefoilan in accordance with the plans prepared by the architect, Mr. G. Moxon. The estimated cost is £20,000.

Lenham.—The Kent County Council have purchased a site at Down Court Manor Farm on which to erect a sanatorium for the county. Mr. E. T. Hall, of 54 Bedford Square, W.C., has been appointed architect for the scheme.

Newtown.—Tenders are invited by the King Edward VII. Welsh National Memorial Association for the erection of a tuberculosis hospital at Newtown, Montgomeryshire. The architects are Messrs. Deakin and Howard Jones, Plas Yynys, Borth, Cardiganshire, from whom full particulars may be obtained.

Nottingham.—The Guardians of the Poor invite tenders for the erection of a children's central home and administrative centre at Hartley Road, Nottingham. Forms of tender, specifications, and bills of quantities may be obtained from the architects, Messrs. Sutton and Gregory, Bromley House, Nottingham.

Summersdale.—The Westhampnett Rural District Council have resolved to acquire a site at Summersdale, at a cost of £225, for the erection of an isolation hospital.

Trowbridge.—The Wiltshire County Council have decided to provide three hospital blocks of twelve beds each for the treatment of advanced phthisis, at an estimated cost of £4,500.

